# Cut-out risk factor analysis after intramedullary nailing for the treatment of extracapsular fractures of the proximal femur: a retrospective study

**DOI:** 10.1186/s12891-022-05054-w

**Published:** 2022-02-01

**Authors:** Jae Youn Yoon, Sehan Park, Taehyun Kim, Gun-Il Im

**Affiliations:** Department of Orthopedic Surgery, Dongguk University Ilsan Hospital, 10326 Goyang, Republic of Korea

**Keywords:** Intertrochanteric fracture, Basicervical femoral neck fracture, Intramedullary nail cut-out, Complication

## Abstract

**Backgrounds:**

The basic method of surgical treatment for extracapsular hip fractures (ECFs), including intertrochanteric fracture and basicervical fracture (BCF), is osteosynthesis. Intramedullary nails are among the most commonly used fixation devices for these fractures. Our study aimed to report the clinical outcomes of ECF treatment with two different nail devices and to analyze the risk factors associated with screw cut-out.

**Methods:**

We retrospectively reviewed the medical records of 273 patients (300 cases) from a single institution who underwent surgical treatment for ECF between January 2013 and October 2018. Overall, 138 patients were eligible for the study and were divided into two groups according to the osteosynthesis device used. We evaluated the clinical outcomes of fracture surgery and performed univariate and multivariate regression analyses to identify risk factors associated with screw cut-out in each group.

**Results:**

We used proximal femoral nails (group 1) to treat 83 patients and cephalomedullary nails (group 2) to treat 55 patients. Nine cut-outs (group 1, 6 cases; group 2, 3 cases) occurred during follow-up. The patients’ high body mass index (BMI) (*p* = 0.019), BCFs (*p* = 0.007), non-extramedullary reduction in the anteroposterior and lateral planes (*p* = 0.032 and *p* = 0.043, respectively), and anti-rotation screw pull-outs (*p* = 0.041) showed a positive correlation to screw cut-out in the univariate analysis of group 1. In group 2, only BCFs was positively correlated (*p* = 0.020). In the multivariate analysis of group 1, the patients’ BMIs (*p* = 0.024) and BCFs (*p* = 0.024) showed a positive correlation with cut-out. Meanwhile, the multivariate analysis of group 2 did not identify any factors associated with cut-out.

**Conclusions:**

The cut-out risk was significantly higher in the BCF cases, regardless of the nail design used. Considerable attention should be paid to treating such unstable fractures. We expect that new-generation nails using a helical blade, or interlocking derotation and interlocking screws may improve surgical outcomes.

## Background

The basic method of surgical treatment for extracapsular hip fractures (ECFs), such as intertrochanteric fractures and basicervical fractures (BCFs) is osteosynthesis [1]. Various fixation devices can be selected according to the fracture pattern, physical characteristics of the instruments, patients’ medical conditions, and surgeons’ preferences. Intramedullary nails (IMNs) are load-sharing devices that are less invasive than dynamic hip screws. For these reasons, we treated most of the ECFs in elderly patients using IMNs [2, 3].

Despite the advantages of IMNs, they are associated with ‘cut-out’, a common and serious complication [4–6]. Cut-out is defined as the collapse of the neck-shaft angle into varus, leading to extrusion of the screw from the femoral head [7]. The incidence varies from 1.8 to 16.5%, depending on the study, and the causes of cut-out are considered multifactorial [8–10]. In this report, (1) we compared the clinical outcomes of cut-out using two different types of IMN for the surgical treatment of ECF in elderly patients, and (2) analyzed clinical risk factors associated with nail cut-out.

## Methods

### Study design and patient selection

**Fig. 1:**
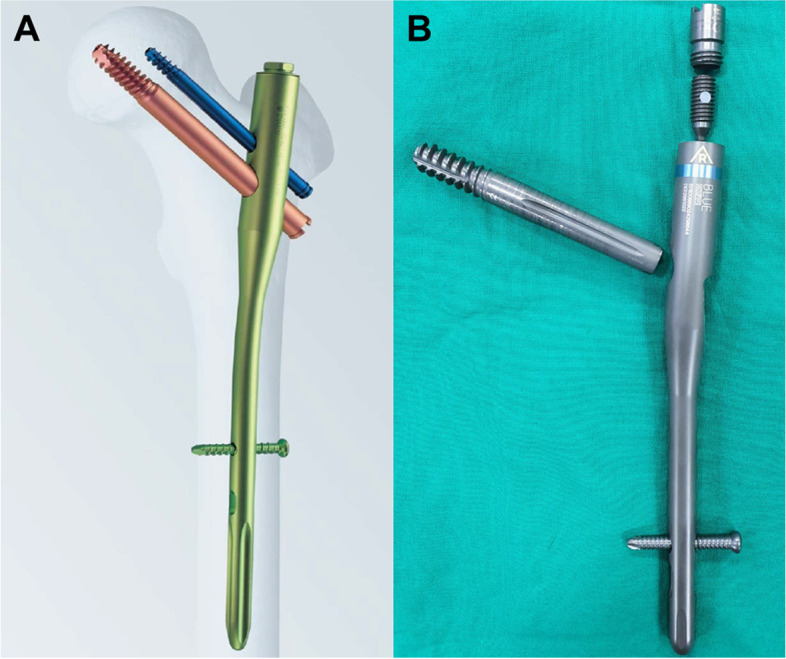
Photograph of two different nail system. **A** Proximal femoral nail (PFN, Synthes, Paoli, Switzerland) and **B** Cephalomedullary nail (CMN, Zimmer, Warsaw, USA)

**Fig. 2:**
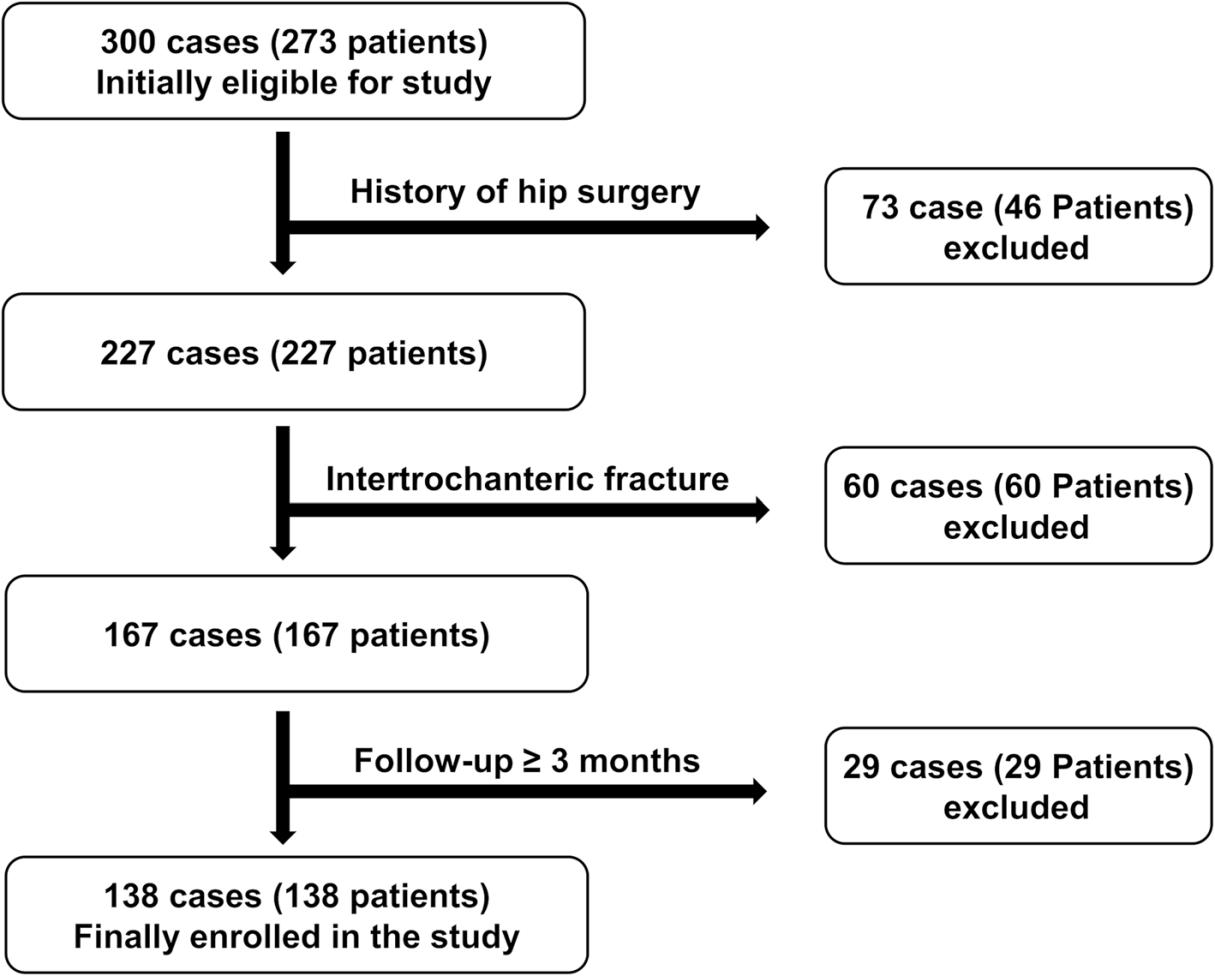
Patient flowchart

 This retrospective study was approved by the relevant institutional review board. We first reviewed patients diagnosed with hip fractures and treated with internal fixation using IMNs at our institution from January 2013 to October 2018. The inclusion criteria were as follows: (1) patients with an intertrochanteric femoral fracture, (2) patients without surgical or interventional histories of the contralateral hip before and after surgery, (3) patients who were followed for a minimum of 3 months after treatment, and (4) patients who were able to walk before surgery. Meanwhile, patients with (1) intracapsular type femur neck fracture (i.e., transcervical or subcapital fracture, basicervical fracture without trochanteric extension) or subtrochanteric fracture, (2) patients with a prior surgical history of the affected hip & contralateral hip before and after surgery, (3) patients who were lost to follow-up or those who expired within three months after surgery, (4) bedridden status or wheelchair-bound patients before surgery were excluded from the study. We then divided the patients into two groups based on the nail device used for fixation. We used the proximal femoral nail (PFN, Synthes, Paoli, Switzerland), which has an anti-rotation screw for additional rotational stability, in earlier periods of the study. Later, we used a cephalomedullary nail (CMN, Zimmer, Warsaw, USA), a simpler device with a single lag screw. Both devices have similar design characteristics, except for the proximal lateralization angle (PFN: 6°, CMN: 4°) and the existence of an anti-rotation pin in PFNs versus an anti-rotation set screw in CMNs (Fig. 1). All patients in both groups were treated using a lag screw to fix the proximal segment, and for patients in the PFN group, the surgery was performed using a dual screw system with an anti-rotation screw. A total of 138 out of 273 patients (300 cases) were finally enrolled in the study based on the inclusion criteria, of which 83 patients were in the PFN group (group 1) and 55 patients were in the CMN group (group 2) (Fig. 2).

### Fracture classification

The fracture pattern was classified by two skilled physicians (JYY and GII) according to the AO Foundation/Orthopaedic Trauma Association classification, which was revised in 2018 [11]. Simple radiographs and 3D computed tomography were used to assess fracture patterns and the presence of a BCF extending to the trochanteric area. We considered a BCF as an ECF only when accompanied by a trochanteric fracture extension [12].

### Surgical procedures and postoperative rehabilitation

**Fig. 3:**
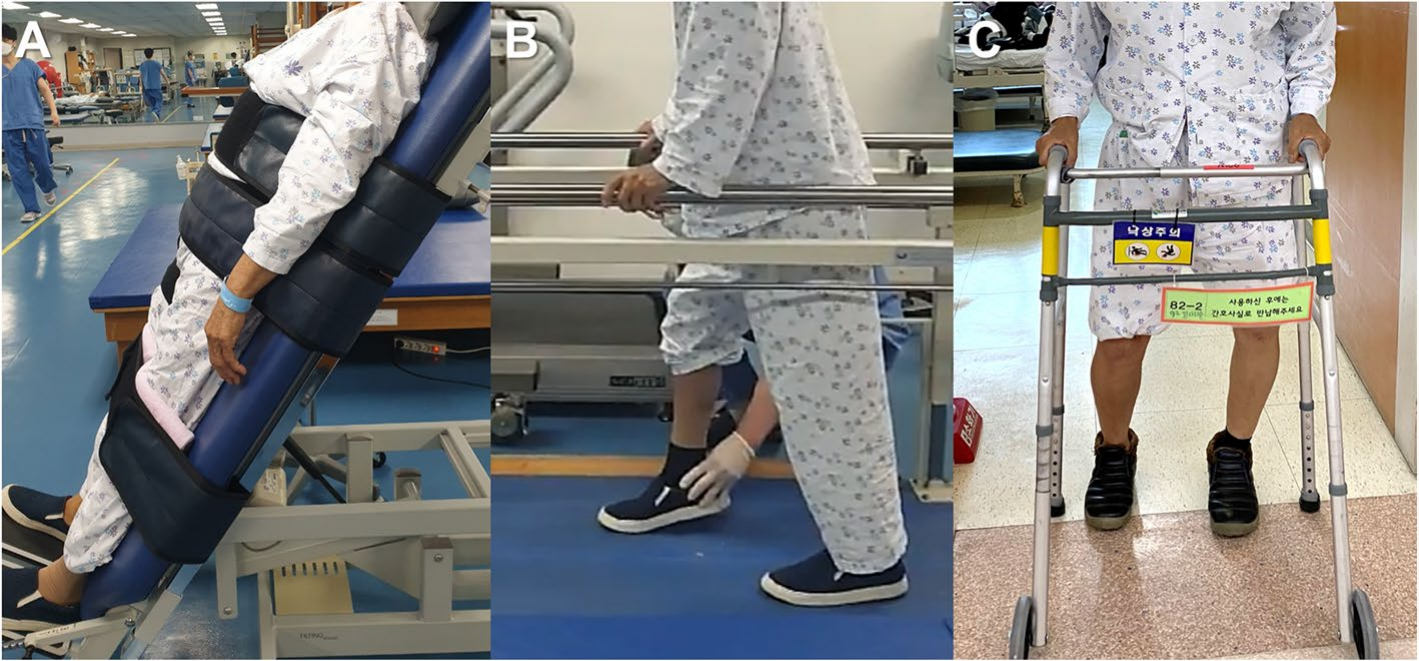
Patient ambulation protocol. **A** Patients first start with tilt table standing at postoperative 1 to 2 days. **B** Parallel bar gait with 30% partial weight-bearing is being sequentially trained after tilt table exercise. **C** A three-point gait with a double-crutch or walker is finally educated and then discharged

All surgical procedures were performed by a single senior surgeon (GII) at our institution. Patients were placed in the supine position on a fracture table, and a c-arm image intensifier was used to assess fracture reduction quality. Most surgeries were performed using the closed reduction technique, but in rare cases of irreducible or unmaintainable fractures, we performed mini-open reduction using a Hohmann retractor or curved Kelly forceps. When acute severe pain subsided, 2 or 3 days after the operation, we trained the patients to start protected weight-bearing (approximately 1/3 of the individual bodyweight) using a walker (Fig. 3). After discharge, patients were followed up at 6 weeks, 3 months, 6 months, and 12 months postoperatively, and then annually.

### Radiologic assessment

The Singh index is a radiographic grading system for osteoporosis and is measured from the normal contralateral hip of the patients [13]. The grade ranges from 1 (principal tensile and compressive trabeculae are markedly reduced or absent) to 6 (all normal trabecular groups are visible), and patients with Singh’s grades ≤3 were considered to have significant osteoporosis. The lag screw positioning was analyzed using the Cleveland index (zones 1–9) [14]. Cleveland index zones 5 (center, center) and 8 (center, inferior) were considered as ideal, and zones 4, 6, 7, and 9 (non-central) were considered as non-ideal. The tip-apex distance was calculated from immediate postoperative X-rays, based on the method suggested by Baumgaertner et al. [7]. Fracture reduction status was evaluated by several measurement methods. Restoration of the neck-shaft angle and axis deviation angle between the femoral neck and the lag screw was measured using anteroposterior radiographs, which were graded as good (<5 varus or valgus), acceptable (5–10), and poor (>10) [7, 15]. Medial and anterior cortical continuity was evaluated in the anteroposterior and translateral views and fracture reduction quality was classified as extramedullary, anatomical, and intramedullary. Finally, postoperative complications, such as surgical site infection (superficial or deep), fracture nonunion, post-traumatic osteonecrosis, fixation failure, screw cut-out, and reoperation of any cause were also investigated.

### Statistical Analysis

Normality and equity of variance were used to assess the differences between the two groups. The metric data were presented as mean values ± 95% confidence intervals (CIs), while categorical data were presented as absolute frequency and percentage distributions. The student’s t-test or the Mann–Whitney U test was used for processing continuous data, and the Chi-square test or the Fisher’s exact test for categorical data. Stepwise selection was performed to control multiple collinearities between independent variables, with an entry condition of *p* < 0.05 and a removal condition of *p* > 0.10. Finally, multivariate logistic regression analysis was performed using variables that were found to be statistically significant (*p* < 0.05) in the univariate analysis.

## Results

We examined 62 female and 21 male patients, with a mean age of 78.3 years (95% CI, 77.0–9.6). Patient demographic data are presented in Table 1, and there were no statistically significant differences in patient demographics. The follow-up duration differed between the two groups, but it was at least 6 months for all patients.

**Table 1:** Demographics and complications for patients in the PFN and CMN groups

Variables, mean value (mean±SD)	All patients (*n* = 138)	PFN group (*n* = 83)	CMN group (*n* = 55)	*p*-value
**Age (years)**	78.3 ± 8.0	77.7 ± 8.5	79.2 ± 7.3	0.419
**Sex (Female : Male)**	102 : 36	62 : 21	40 : 15	0.796
**BMI (kg/m** ^2^ **)**	21.6 ± 3.9	21.1 ± 3.9	22.4 ± 3.7	0.232
**Follow-up period (months)**	19.4 ± 22.5	25.3 ± 26.5	10.5 ± 9.0	0.005
**Time of surgical delay (days)**	3.2 ± 4.0	3.2 ± 3.7	3.1 ± 3.9	0.775
**Preoperative KOVAL grade**	1.9 ± 1.4	2.0 ± 1.2	1.8 ± 1.5	0.288
**Medical comorbidity**				
DM	40	25	15	0.718
HTN	78	48	30	0.703
ESRD	4	1	3	0.301
COPD or ILD	5	4	1	0.648
Stroke	31	18	13	0.788
Dementia	6	2	4	0.216
**AO/OTA classification**				0.233
A1	100	64	36	
A2	30	14	16	
A3	8	5	3	
**Basicervical fracture extension**	22	14	8	0.715
**Postoperative complications**				
Wound infection	3	1	2	0.563
Nonunion	5	3	2	1.000
ONFH	2	1	1	1.000
Screw cut-out	9	6	3	1.000

*AO/OTA *AO Foundation/Orthopaedic Trauma Association classification, *BMI *body mass index, *CI* confidence interval, *CMN* cephalomedullary nail, *COPD* chronic obstructive pulmonary disease, *DM* diabetes mellitus, *ESRD* end-stage renal disease, *F* female, *HTN* hypertension, *ILD* interstitial lung disease, *M* male, *ONFH* osteonecrosis of femur head, *PFN* proximal femur nail, *SD* standard deviation

The incidences of postoperative complications, including wound infection, fracture nonunion, osteonecrosis of the femoral head, and nail perforation, in both groups are presented in Table 1. Fracture nonunion occurred in 12 patients (8.69%), of which 9 patients (6.52%) experienced nail perforation during follow-up. A serial radiographic assessment of 9 cases with nail perforation showed that all cases involved implant cut-out, and no cases involved cut-through. In the PFN group only, there were 8 cases (7.23%) of anti-rotation screw pull-out during follow-up.

We performed a univariate logistic regression analysis of each group to verify the correlation between the patients’ demographic factors, radiologically measured parameters, and implant cut-out. Table 2 shows the results of the univariate logistic regression analysis of the PFN group. The patients’ high body mass index (BMI) (*p* = 0.019) and presence of BCF (*p* = 0.005) were positively correlated with the incidence of implant cut-out. For the radiologic parameters, the absence of cortical overlap between the fragments in the anteroposterior (*p* = 0.032) and lateral planes (*p* = 0.043) showed significant correlation with implant cut-out. In the analysis of the CMN group, only the presence of BCF showed a statistically significant positive correlation (*p* = 0.020), and all other factors did not show a statistically significant correlation (Table 3).

**Table 2:** Univariate analysis of variables associated with screw cut-out in PFN group

	All patients (*n* = 83)	No Cut-out (*n* = 77)	Cut-out (*n* = 6)	*p*-value	Odds ratio	95% Cl
**Age at surgery**	78.3	77.4	83.7	0.316	1.06	0.95–1.19
**Sex**				0.330		
Female	62 (74.7%)	56	6			
Male	21 (25.3%)	21 (100%)	0			
**BMI (kg/m** ^2^ **)**	21.1	20.8	25.1	0.019	1.30	1.04–1.62
**Days to Surgery**	3.2	3.1	3.6	0.554	1.05	0.89–1.24
**Fracture classification**				0.519		
AO/OTA A1	64 (77.1%)	60 (93.75%)	4 (6.25%)			
AO/OTA A2	14 (16.8%)	12 (85.7%)	2 (14.3%)			
AO/OTA A3	5 (6.1%)	5 (100%)	0			
**Basicervical fracture extension**				0.007	13.40	2.17–82.93
No	69 (83.1%)	67 (97.1%)	2 (2.9%)			
Yes	14 (16.9%)	10 (71.4%)	4 (28.6%)			
**Singh index**				0.658		
<=3	54 (66.3%)	49 (94.4%)	5 (5.6%)			
>3	29 (33.7%)	28 (96.6%)	1 (3.4%)			
**Cleveland index**				0.549		
Ideal (zone 5, 8)	49 (59.0%)	44 (89.7%)	5 (10.3%)			
Non-ideal (zone 4, 6, 7, 9)	34 (41.0%)	33 (97.1%)	1 (2.9%)			
**Tip-Apex distance (mm)**	14.5	14.5	13.6	0.645	0.94	0.74–1.21
**Neck-shaft angle**				0.300		
Good (<5 degrees)	39 (47.0%)	38 (97.4%)	1 (2.6%)			
Acceptable (5–10 degrees)	25 (30.1%)	22 (88.0%)	3 (12.0%)			
Poor (>10 degrees)	19 (22.9%)	17 (89.5%)	2 (10.5%)			
**Neck-screw axis**				0.248		
Good (<5 degrees)	60 (72.3%)	57 (95.0%)	3 (5.0%)			
Acceptable (5–10 degrees)	17 (20.5%)	15 (88.2%)	2 (11.8%)			
Poor (>10 degrees)	6 (7.2%)	5 (83.3%)	1 (16.7%)			
**Reduction quality (AP view)**				0.032		
Extramedullary	34 (41.0%)	33 (94.3%)	1 (5.7%)			
Anatomical	48 (57.8%)	44 (91.7%)	4 (8.3%)			
Intramedullary	1 (1.2%)	0	1 (100%)			
**Reduction quality (axial view)**				0.043		
Extramedullary	18 (21.7%)	18 (100%)	0			
Anatomical	46 (55.4%)	44 (95.6%)	2 (4.4%)			
Intramedullary	19 (22.9%)	15 (78.9%)	4 (21.1%)			
**Anti-rotation screw pull-out**	8 (9.6%)	6 (75.0%)	2 (25.0%)	0.041	5.92	0.89–39.20

*BMI* body mass index, *CI* confidence interval, *PFN* proximal femur nail

**Table 3:** Univariate analysis of variables associated with screw cut-out in CMN group

	All patients (*n* = 55)	No Cut-out (*n* = 52)	Cut-out (*n* = 3)	*p*-value	Odds ratio	95% Cl
**Age at surgery**	79.2	78.9	83.7	0.235	1.13	0.92–1.38
**Sex**				0.554		
Female	40 (72.7%)	37 (92.5%)	3 (7.5%)			
Male	15 (27.3%)	15 (100%)	0			
**BMI (kg/m** ^2^ **)**	22.4	22.6	19.3	0.136	0.77	0.55–1.09
**Days to Surgery**	3.1	3.1	2.3	0.741	0.91	0.53–1.56
**Fracture classification**				0.410		
AO/OTA A1	35 (63.6%)	34 (97.1%)	1 (2.9%)			
AO/OTA A2	15 (27.3%)	13 (86.7%)	2 (13.3%)			
AO/OTA A3	5 (9.1%)	5 (100%)	0			
**Basicervical fracture extension**				0.020	0.10	0.04–0.22
No	47 (85.5%)	47 (100%)	0			
Yes	8 (14.5%)	5 (62.5%)	3 (37.5%)			
**Singh index**				1.000		
<=3	33 (60.0%)	31 (93.9%)	2 (6.1%)			
>3	22 (40.0%)	21 (95.5%)	1 (4.5%)			
**Cleveland index**				1.000		
Ideal (zone 5, 8)	52 (94.5%)	49 (94.2%)	3 (5.8%)			
Non-ideal (zone 4, 6, 7, 9)	3 (5.5%)	3 (100%)	0			
**Tip-Apex distance (mm)**	16.4	16.2	19.2	0.213	1.21	0.90–1.63
**Neck-shaft angle**				0.208		
Good (<5 degrees)	22 (40%)	22 (100%)	0			
Acceptable (5–10 degrees)	23 (41.8%)	20 (87.0%)	3 (13.0%)			
Poor (>10 degrees)	10 (18.2%)	10 (100%)	0			
**Neck-screw axis**				0.440		
Good (<5 degrees)	36 (65.5%)	34 (94.4%)	2 (5.6%)			
Acceptable (5–10 degrees)	12 (21.8%)	12 (100%)	0			
Poor (>10 degrees)	7 (12.7%)	6 (85.7%)	1 (14.3%)			
**Reduction quality (AP view)**				0.568		
Extramedullary	18 (32.7%)	18 (100%)	0			
Anatomical	36 (65.5%)	33 (91.7%)	3 (8.3%)			
Intramedullary	1 (1.8%)	1 (1.9%)	0			
**Reduction quality (Axial view)**				0.330		
Extramedullary	4 (7.3%)	4 (7.7%)	0			
Anatomical	38 (69.1%)	37 (71.2%)	1 (33.3%)			
Intramedullary	13 (23.6%)	11 (21.2%)	2 (66.7%)			

*BMI* body mass index, *CI* confidence interval, *CMN* cephalo-medullary nail

For the PFN group, we also conducted a multivariate regression analysis of the above four factors that showed a significant correlation in the univariate analysis (Table 4), and we found that BMI (*p* = 0.024) and BCF (*p* = 0.024) had a statistically significant positive correlation with nail cut-out.

**Table 4:** Multivariate analysis of variables associated with screw cut-out in PFN group

	Beta coefficient	Standard Error	*p*-value	Odds ratio	95% CI
**BMI (kg/m** ^2^ **)**	0.477	0.211	0.024	1.611	1.066–2.436
**Basicervical fracture extension**					
No					
Yes	5.133	2.282	0.024	169.450	1.935–1483.447
**Reduction quality (Axial view)**					
Extramedullary			0.205		
Anatomical	17.119		0.998	0.000	.
Intramedullary	20.906		0.998	0.000	.
**Anti-rotation screw pull-out**	3.661	1.960	0.062	38.904	0.835–1812.430
*BMI* body mass index, *CI* confidence interval, *PFN* proximal femur nail					

**Fig. 4:**
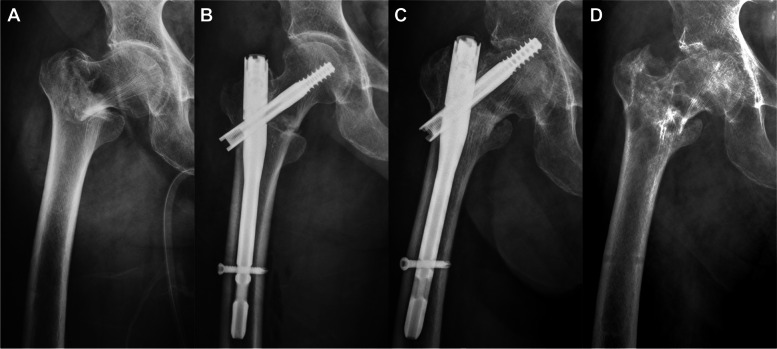
Eighty-seven-year-old female after fall down injury. **A** Anteroposterior X-ray image of right hip showing an extracapsular fracture (basicervical type). **B** The patient was surgically treated with cephalomedullary nail **C** But prominent cut-out was identified at postoperative 4.2 months follow-up. **C** Simple instrument removal was performed due to the patient’s poor general condition

The main postoperative complications are presented in Table 1. We performed implant removal in 2 patients and screw exchange in 3 patients and converted to arthroplasty in 7 patients. The mean times to reoperation were 9, 13.4 months, and 16.2 months, respectively. We removed the implant in 2 cut-out cases: in 1 patient whose general medical condition did not allow revision arthroplasty despite the prominent cut-out and fracture nonunion (Fig. 4), and in another patient whose fracture healed completely without damaging the acetabular cartilage. Screw exchange was planned for 3 patients whose cut-outs were radiologically confirmed during the fracture-healing process. No notable articular damage was identified at the time of reoperation, and all patients achieved complete bone union by the final follow-up. Of the 7 patients who switched to arthroplasty, 5 cases were attributable to fracture nonunion (2 cases accompanying implant cut-out) and 2 cases to post-traumatic osteonecrosis of the femoral head. Despite the need for surgical correction, 2 cut-out patients were put on hold because of poor general health conditions or underlying medical comorbidities.

## Discussion

The overall cut-out rate in our study, with a mean follow-up of 19.4 months, was 6.52% (9 out of 138 patients). Since the incidence of implant cut-out after surgery for intertrochanteric fractures varies from 1.85 to 16.5%, our results are comparable to those of other reports [16, 17]. Several factors have been suggested as risk factors for implant cut-out in previous studies. Bojan et al. reported that reduction status, screw position, fracture pattern, and implant design are major risk factors for implant cut-out [8]. Morvan et al. also stated that tip-apex distance and reduction quality are closely related to cut-out [5]. However, the patient’s age, fracture pattern, and degree of osteoporosis were not associated with the complication.

In our univariate and multivariate logistic regression analyses, BCF was the most significant risk factor for screw cut-out. BCF is a relatively rare and controversial fracture that occurs at a junction between the femoral neck and intertrochanter region [12]. It is reported to have a relatively higher fixation failure and reoperation rate due to higher angular and rotational instability compared to an intertrochanteric femur fracture [18–20]. There is still no proper fracture classification system for BCFs, and there is an ongoing controversy over appropriate implant selection and surgical options [19]. Traditionally, the use of compression hip screws was recommended for BCFs, but recently, with IMN design improvements, such as the introduction of a helical blade and dual lag screw system, more favorable clinical results have been reported using IMNs [20]. Based on the study by Saarenpää et al., which differentiated the BCF based on the trochanteric extension of fractures, we defined BCF with trochanteric extension as ECF and performed osteosynthesis using IMNs [12].

In addition to the presence of BCF, the patient’s high BMI, poor reduction quality (no cortical overlap), and early pull-out of anti-rotation screws also correlated with the cut-out in the PFN group. Regarding the higher BMI in the cut-out group, 3 of the 6 patients had preceding neuromotor comorbidities (5 out of 77 patients in the control group), such as Parkinson’s disease or cerebral stroke/hemorrhage sequelae. Due to the diversity of the patient’s underlying medical history, the presence of neuromotor disorders was not applied as a variable for regression analysis. However, we believe that the patients’ difficulty following routine rehabilitation protocols and performing weight-restricting exercise would have resulted in fixation failure and, consequently, screw cut-out.

Pull-out of the anti-rotation screw is a unique complication applied only to dual screw systems, such as PFNs. As the PFN system does not have a set screw to control the rotation or sliding of screws, is highly dependent on the patient’s cancellous bone quality [17, 21]. Rotational and angular instability related to fracture type and reduction status may also result in postoperative screw loosening or sliding. We radiologically identified anti-rotation screw loosening in 8 of 83 patients, and cut-out was confirmed in 2 patients. All 6 patients without screw cut-out were initially classified as having simple pertrochanteric fractures (31A1.2; 5 cases and 31A1.3; 1 case) based on the AO Foundation/Orthopaedic Trauma Association classification system. Although excessive early screw pull-out occurred at 4 and 7 weeks after surgery in 2 patients, the fractures healed well without further displacement. Meanwhile, 2 cut-out cases were caused by unstable intertrochanteric fractures (31A2.2; 1 case and 31A2.3; 1 case), and both cases were confirmed to have BCF. No cases of cut-out occurring due to the ‘z-effect’ or ‘reverse z-effect’ were identified in the PFN group [17, 22].

Our study is first limited by its non-randomized study design and no functional outcome, and the small number of enrolled patients. To compensate for this limitation, all patients were treated by a single surgeon (GII) at a single center. Meanwhile, lack of functional scores does not undermine the strength of this study, since the main purpose of this study is to radiologically evaluate postoperative complications such as screw cut-out, nonunion, and osteonecrosis. Second, there may be a selection bias due to the study exclusion criteria, such as a surgical history of the contralateral hip and the minimum follow-up period. However, as many previous studies have set similar criteria to accurately analyze clinical prognoses after hip surgery, the reliability of our results is not undermined [23, 24].

## Conclusions

Our study identified several factors associated with screw cut-out after internal fixation of ECFs. The presence of a BCF was the most significant risk factor for postoperative screw cut-out, regardless of the fixation device used. Several studies have recently reported favorable clinical results using improved nail designs, such as a rotation-controlled lag screw with a U-clip, a helical blade, and twin interlocking derotation and compression screws [15, 25–27]. Large-scale randomized prospective studies are expected to verify the clinical efficacy and superiority of various fixation devices in treating such fractures.

## Data Availability

The datasets used and analyzed during the current study are available from the corresponding author, as a supplementary file, on reasonable request.
